# Synopsis of the Argentinian scarab genus *Pseudogeniates* Ohaus (Coleoptera, Scarabaeidae, Rutelinae)

**DOI:** 10.3897/zookeys.241.3802

**Published:** 2012-11-13

**Authors:** Mary Liz Jameson, Federico Ocampo

**Affiliations:** 1 Wichita State University, Wichita, Kansas, USA; 2 Instituto Argentino de Investigaciones de Zonas Áridas - Instituto de Ciencias Básicas, CCT-CONICET Mendoza, Argentina

**Keywords:** Endemic, Pampa, Monte, Chaco, intraspecific variation

## Abstract

The scarab beetle genus *Pseudogeniates* Ohaus (Scarabaeidae: Rutelinae: Rutelini) is endemic to Argentina. The genus includes three species: *Pseudogeniates cordobaensis* Soula, *Pseudogeniates intermedius* Ohaus, and *Pseudogeniates richterianus* Ohaus. We characterize the genus, provide a key to species, redescribe and diagnose each species, provide spatial and temporal distributions, and discuss distributions of the species in relation to eco-regions and areas of endemism in Argentina.

## Abstract (es)

El género de escarabajo *Pseudogeniates* Ohaus (Scarabaeidae: Rutelinae: Rutelini) es endémico de Argentina. El género incluye tres especies: *Pseudogeniates cordobaensis* Soula, *Pseudogeniates intermedius* Ohaus, y *Pseudogeniates richterianus* Ohaus. En este trabajo caracterizamos el género, proveemos una clave para las especies, redescribimos y proveemos diagnosis para cada una de ellas y datos de su distribución temporal y espacial y discutimos aspectos de las distribuciónes de las especies en relación de las regiones eco-regiones y áreas de endemismo en Argentina.

## Introduction

**Figures 1–3.**
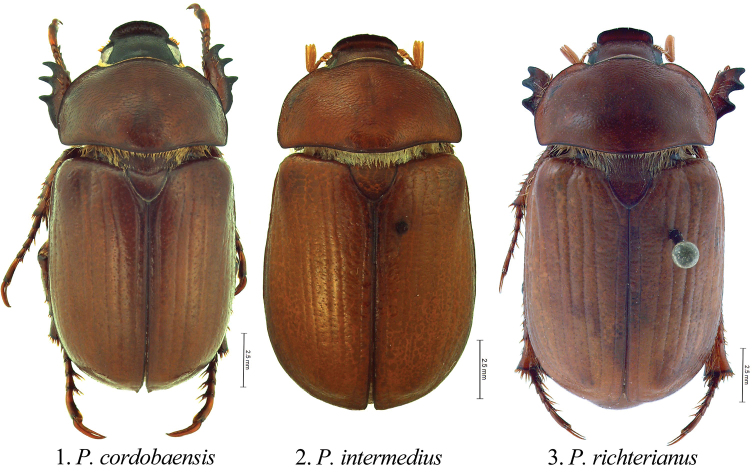
Dorsal habitus of *Pseudogeniates* species **1** *Pseudogeniates cordobaensis* **2** *Pseudogeniates intermedius* **3** *Pseudogeniates richterianus*

The leaf chafer genus *Pseudogeniates* Ohaus (Rutelinae: Rutelini) (Figs 1–3) is endemic to Argentina. Species in the genus are associated with arid habitats in the Pampa, Espinal, Monte, and Chaco eco-regions (Fig. 22) (eco-regions as in Ponce et al. 2002). *Pseudogeniates* species are moderate-sized (12–19 mm), elongate-oval, brown scarabs with striate elytra (Figs 1–3). In many respects, species in the genus are similar in overall gestalt to species in the genus *Geniates* Ohaus (Rutelinae: Geniatini) or drab-colored species in the genus *Anomala* Samouelle (Rutelinae: Anomalini). Actually, however, form of the labrum, elytra, protibia and tarsomeres, and position of the terminal spiracle, place the genus in the tribe Rutelini (Rutelinae) (see “Classification”).Female gender bias in the most wide-spread species and the fact that specimens are rarely encountered in collections have both contributed to the difficulty in circumscribing species in the genus. Our work revealed a high degree of intraspecific variation that may be due to spatial or temporal isolation of populations. Thus, in addition to rarity and sex bias, circumscription of species is complicated by variation in character states. Heretofore, there has been no comprehensive review of species in the genus. This research provides a synopsis of the three species in the genus and information on the distribution of these poorly studied species.

## Taxonomic history

Ohaus (1910) named the genus *Pseudogeniates* for one species, *Pseudogeniates richterianus* Ohaus, and he based the description on female specimens alone. He puzzled over classification of the genus, discussing its affinities with the ruteline tribes Geniatini and Anoplognathini. Both of these tribes are orthochilous rutelines, that is, they belong to a broad group of Rutelinae in which the mouthparts (specifically labrum and mentum) project vertically with respect to the head (Ohaus 1918, 1934; Machatschke 1965; Jameson and Hawkins 2005). Based on overall gestalt and proportions of the “hind body”, Ohaus (1910) compared *Pseudogeniates richterianus* with *Geniates barbatus* Kirby and *Pseudogeniates cylindricus* Burmeister (both Geniatini from South America). He also compared the genus with *Saulostomus weiskei* Ohaus and *Pseudogeniates felschei* Ohaus (both Anoplognathini from Australia) based on the form of the mouthparts. The form of the clypeus and mouthparts were so unusual that when Ohaus first saw specimens, he “believed that the animal was crippled” (Ohaus 1910: 179). After studying two additional female specimens from a different locality, Ohaus realized that these peculiarities were not teratological. Despite lacking male specimens, he described *Pseudogeniates richterianus*, but he declined placing the new genus and species in a ruteline tribe, stating that this would require additional characters from male specimens(Ohaus 1910).

After obtaining additional specimens of *Pseudogeniates* and making comparisons with other Argentinian fauna, Ohaus (1914) placed the genus in the tribe Rutelini (Rutelinae), a tribe of homalochilous rutelines that is characterized by the labrum that is horizontally produced with respect to the clypeus. He discussed affinities of *Pseudogeniates* with *Homonyx* Guerin and *Parhomonyx* Ohaus, both of which are distributed in southern South America and both members of the subtribe Pelidnotina. Based on both male and female specimens, Ohaus (1914) described a new species, *Pseudogeniates intermedius*. Ohaus characterized the two species using differences in the form of the clypeus and antennae: *Pseudogeniates richterianus* possessed nine-segmented antennae (thus differing from all other Rutelini) and *Pseudogeniates intermedius* possessed ten-segmented antennae (the hypothesized ancestral state within the Rutelini). The antennal character state in *Pseudogeniates richterianus* make this species an exception in the key to tribes of Rutelinae (see Jameson 1990, 2005). However, Ohaus did not have a broad enough sampling of specimens of *Pseudogeniates richterianus* to understand the intraspecific variation of this character: antennae are either 9- or 10-segmented in *Pseudogeniates richterianus*. This variation has confounded identification of *Pseudogeniates* species.

The *Genera Insectorum* on the Rutelini (Ohaus 1934) languished for more than 20 years before publication. Realizing the great delay, Ohaus (1915) published his concepts of higher taxa and descriptions of genera. He formalized use of the subtribe Pelidnotina (as “Pelidnotinorum”) and commented on evolution and affinities of *Pseudogeniates*, *Parhomonyx*, and *Homonyx*. He considered *Parhomonyx* to be an “intermediate stage” that “led *Homonyx* to *Pseudogeniates*” (Ohaus 1915: 258), and that characters of the coloration, clypeus, mouthparts, elytra, hind tibia, and antennae indicated these relationships (or this progression of forms). He thought that these taxa were a good example of how differences in rainfall (dry versus wet; e.g., Ponce et al. 2002) and differences in habitat (forest versus steppe) produced adaptations and changes in morphological characters. He also stated that the Argentinian fauna offered a number of examples of these evolutionary transformations, particularly in scarab beetles.

Nearly 100 years after Ohaus’ work on *Pseudogeniates*, Soula (2009) reviewed the genus based on six specimens in the Ohaus collection (housed at ZMHB), described a new species, *Pseudogeniates cordobaensis*, based on specimens from the type series of *Pseudogeniates intermedius* (which included three males and one female specimen), described the type specimens for each of the species of *Pseudogeniates*, and commented briefly on the unusual character states of the genus. Soula’s species descriptions are not comparative; they do not allow separation and identification of species in the genus; and, because they are based on a limited number of specimens, they do not take into account variation within the species. In addition, Soula (op. cit.) did not provide a key to species and diagnoses. For these reasons, Soula’s work is of little utility for identification and understanding of biodiversity of this group.

### Definition of taxonomic characters and character examination

This research is based on 56 specimens from collections including type specimens. About one third of these specimens (19 specimens) were identified in collections; the remaining two thirds were not identified. Out of the 19 specimens that were identified, 9 were incorrectly identified, 4 were correctly identified, and 6 were type specimens. Two specimens were incorrectly identified to genus. Specimens for this research are deposited at the CMNC (Canadian Museum of Nature Collection, Ottawa, Canada), FMNH (Field Museum of Natural History, Chicago, Illinois, USA); IAZA (Instituto Argentino de Investigaciones de Zonas Áridas, Mendoza, Argentina); MACN (Museo Argentino de Ciencias Naturales, Buenos Aires, Argentina); MLJC (Mary Liz Jameson collection, Wichita, Kansas, USA); UCCC (Universidad de Concepción, Concepción, Chile); USNM (United States National Collection, Washington, D.C., USA); and ZMHB (Museum für Naturkunde der Humboldt Universitat zu Berlin, Berlin, Germany).

Morphological characters formed the basis of this work. The broadest range of potentially phylogenetically informative morphological characters was used for morphological analyses and comparisons. For measurements, we used an ocular micrometer. Body measurements, puncture density, puncture size, and density of setae are based on the following standards. Body length was measured from the apex of the clypeus to the apex of the pygidium. Body width was measured at the widest width of the elytra. Puncture density was considered ‘dense’ if punctures were nearly confluent to less than two puncture diameters apart, ‘moderately dense’ if punctures were from two to six puncture diameters apart, and ‘sparse’ if punctures were separated by more than six puncture diameters. Puncture size was defined as ‘small’ if punctures were 0.02 mm in diameter or smaller; ‘moderate’ if 0.02–0.07 mm, ‘moderately large’ if 0.07–0.12 mm, and ‘large’ if 0.12 mm or larger. Setae density was defined as ‘dense’ if the surface was not visible through the setae, ‘moderately dense’ if the surface was visible but with many setae, and ‘sparse’ if there were few setae. It should be noted that setae are subject to wear and may be abraded away. Elytral discal striae are defined as the striae located between the elytral suture and the elytral humerus. The interocular width measures the number of transverse eye diameters that span the width on the frons between the eyes. This was measured by placing the ocular micrometer in a position such that it intersects the frons and eyes (dorsal view), focusing on the surface of the frons, and then measuring the width of the frons and width of the eyes without adjusting the focus. Mouthparts, wings, and genitalia were examined and card-mounted beneath the specimen. Some specimens were quite fatty, with internal and external greasy build-up. These specimens were cleaned in acetone prior to dissection.

Characters and specimens were observed with 6-48× magnification and fiber-optic illumination. Digital images of specimens and structures were captured using the Leica Application Suite V3.8. Images were edited in Adobe Photoshop CS2 (background removed, contrast manipulated).

Species are characterized by combinations of characters including the form of the mentum and maxilla, form of the metacoxa, and form of the ventral plate of the male parameres. We use the phylogenetic species concept (Wheeler and Platnick 2000) in this work: “A species is the smallest aggregation of (sexual) populations or (asexual) lineages diagnosable by a unique combination of character states.”

Specimen localities were translated into latitude and longitude using GoogleEarth (http://www.google.com/earth/index.html ). Maps were generated by entering these data into Microsoft Excel 2008 and uploaded to EarthPoint (http://www.earthpoint.us/ExcelToKml.aspx ) and GoogleEarth (Supplementary File: Pseudogeniates Locality Table.xls). These tools allow for interactive mapping and addition of data by subsequent users. Description of Argentinian eco-regions follows Ponce et al. (2002). Argentinian areas of endemism follow Cabrera and Willink (1973) and Szumik et al. (2012).

#### Pseudogeniates

Ohaus, 1910

http://species-id.net/wiki/Pseudogeniates

Figs 1 [Fig F2] [Fig F3] [Fig F4] [Fig F5] –22

Pseudogeniates Ohaus 1910: 179–180

##### Type species.

*Pseudogeniates richterianus* Ohaus 1910: 180. By monotypy.

##### Tribal classification.

The genus *Pseudogeniates* is a member of the tribe Rutelini. In overall appearance, however, species in the genus *Pseudogeniates* are similar to species in the genera *Geniates* (Geniatini) and *Anomala* (Anomalini). Species in the genus *Pseudogeniates* can be separated from both of these tribes based on the margin of the elytra that lacks an obvious membranous border (membranous border present at the elytral apex in both Geniatini and Anomalini). Additional characters that separate the Rutelini and Geniatini include: the labrum that is horizontally produced with respect to the clypeus in the Rutelini (vertically produced in the Geniatini) and protarsomeres that are subcylindrical and lacking ventral setose pads (dorsoventrally flattened and densely setose ventrally in the Geniatini). Additional characters that separate the Rutelini and Anomalini include: protibia with inner, protibial spur apical in the Rutelini (inner, apical spur subapical in Anomalini) and terminal spiracle positioned in pleural suture in the Rutelini (terminal spiracle not positioned in pleural suture in Anomalini). For a key to tribes of Rutelinae, see Jameson (1990, 2005).

##### Subtribal classification.

Ohaus (1915, 1934) placed the genus *Pseudogeniates* in the tribe Rutelini and subtribe Pelidnotina. Based on morphological data, this subtribe was demonstratively paraphyletic and it was eliminated (Jameson 1998). Soula (2009), without justification or discussion, continued use of this higher-level taxon for the genus. We consider the genus *Pseudogeniates* to be a member of the tribe Rutelini (without subtribal designation).

##### Phylogeny.

Sister group relationships have not been examined for the genus or for species within the genus.

##### Diagnosis of adults.

**Figures 4–10. F2:**
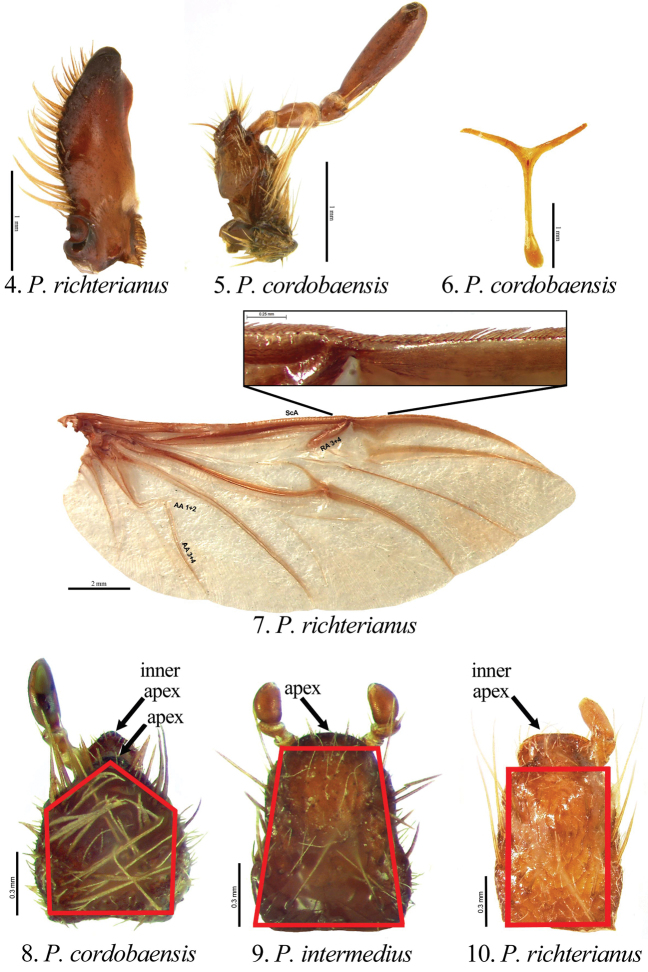
Characters for species of *Pseudogeniates* **4** Left mandible of *Pseudogeniates richterianus*, dorsal view (with one apical, acute, recurved tooth; poorly developed scissorial region, and narrow molar region) **5** Maxilla of *Pseudogeniates cordobaensis*, ventral view (lacking teeth, terminal segment of palpus with dorsal, longitudinal sulcus) **6** Spiculum gastrale of *Pseudogeniates cordobaensis* **7** Wing of *Pseudogeniates richterianus* showing form and inset showing dense, thick setae associated with ScA and region anterior to RA3+4 **8** Mentum, ventral view, of *Pseudogeniates cordobaensis* (shape pentagonal, inner apex projecting anteriorly and with inner shelf) **9** Mentum, ventral view, of *Pseudogeniates intermedius* (shape broadly trapezoidal, apex not projecting anteriorly and without inner shelf) **10** Mentum, ventral view, of *Pseudogeniates richterianus* (shape rectangular, inner apex projecting anteriorly and without inner shelf)

**Figures 11–15. F3:**
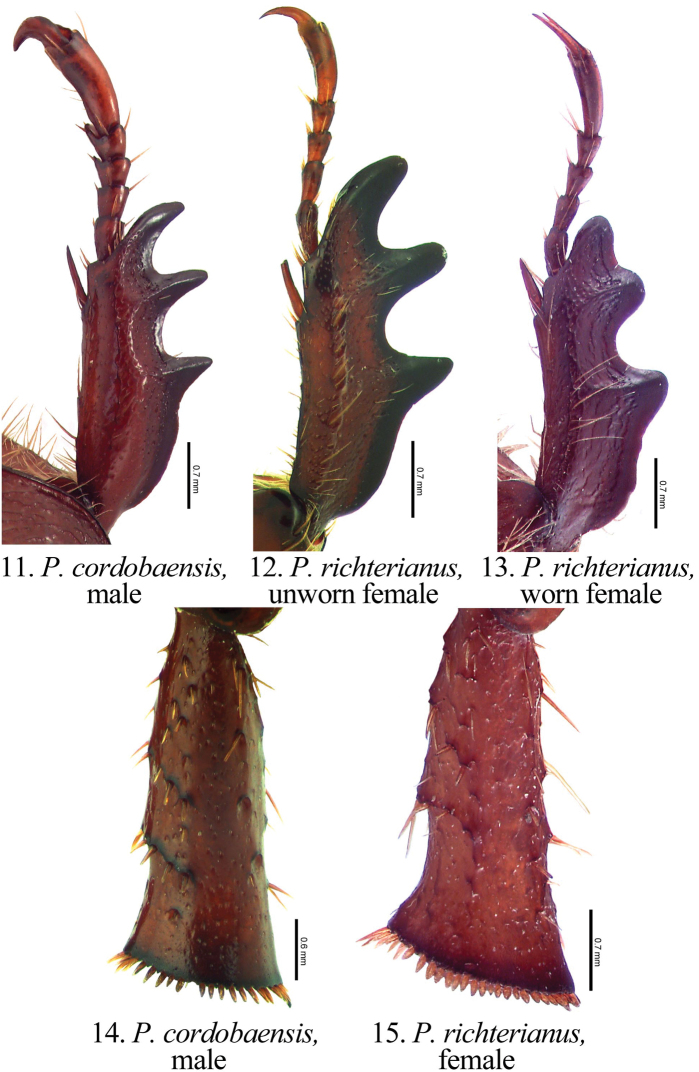
Form of protibia (**11–13**) and metatibia (**14–15**) in *Pseudogeniates* species **11** Protibia of *Pseudogeniates cordobaensis* (male) **12** Protibia of unworn specimen of *Pseudogeniates richterianus* (female) showing **13** Protibia of worn specimen of *Pseudogeniates richterianus* (female) **14** Metatibia of *Pseudogeniates cordobaensis* (male) **15** Metatibia of *Pseudogeniates richterianus* (female)

**Figures 16–18. F4:**
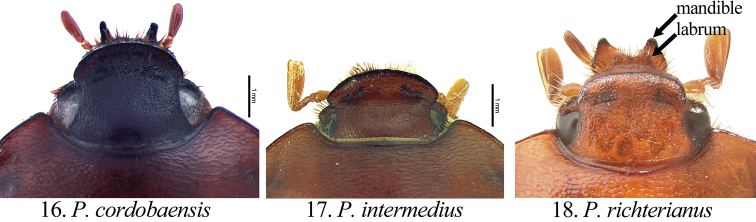
Form of the head (dorsal view) in *Pseudogeniates* species **16** *Pseudogeniates cordoboaensis* showing form of clypeal apex **17** *Pseudogeniates intermedius* showing form of clypeal apex **18** *Pseudogeniates richterianus* showing form of clypeal apex, labrum, and mandible

Members of the genus *Pseudogeniates* differ from other genera in the tribe Rutelini by the following combination of characters: feathery fringe of setae on the ventral edge of the elytra present; elytra obviously striate (Figs 1–3); mesosternal peg lacking; claws simple on all legs (not toothed) (Figs 11–13); frontoclypeal suture incomplete (Figs 16–18); clypeal apex broadly reflexed (Figs 16–18); apex of labrum extending beyond clypeal apex, visible from dorsal view (Fig. 18); apex of mandible with one, apical, recurved tooth (Figs 4, 18); maxillary teeth lacking (Fig. 5).

##### Similar taxa.

Species in the genus *Pseudogeniates* share several characters with *Parhomonyx fuscoaeneus* Ohaus, a monotypic taxon that is also endemic to southern South America. The following characters are shared: fringe of setae at apex of elytra, mesosternal process lacking, mandible with one external tooth, elytra striate, and claws simple. However, *Pseudogeniates* differs from *Parhomonyx* based on the external margin of the mandible that is straight (external margin lobe-like in *Parhomonyx*), maxillary teeth lacking (maxilla with well developed teeth in *Parhomonyx*), maxillary palp rod-shaped (broadly elliptical in *Parhomonyx*), and fifth tarsomere on all legs of males and females lacking an internal tooth (with two well developed internal teeth on the fifth meso- and metatarsomeres of males and females of *Parhomonyx*).

##### Description of adults.

**Figures 19–21. F5:**
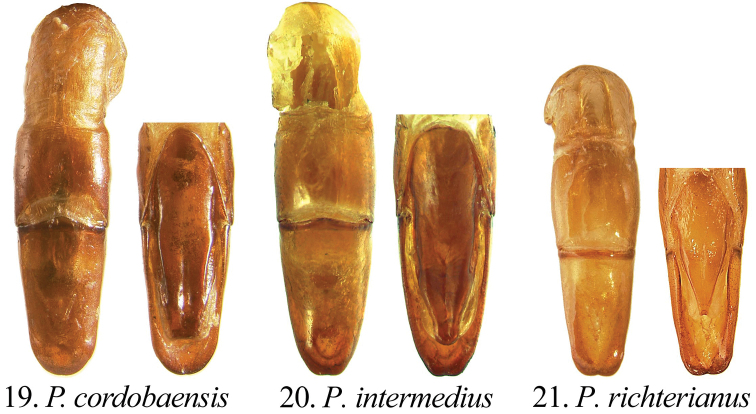
Form of male parameres (dorsal and ventral views) in *Pseudogeniates* species **19** *Pseudogeniates cordobaensis* (ventral plate nearly as long as dorsal plate, apex quadrate) **20** *Pseudogeniates intermedius* (ventral plate nearly as long as dorsal plate, apex rounded) **21** *Pseudogeniates richterianus* (ventral plate about half length of dorsal plate, apex acute)

Length from apex of clypeus to apex of pygidium 12.0–19.0 mm; width at mid-elytra 6.0–11.0 mm. Color: Dorsal and ventral surfaces testaceous to castaneous. Form (Figs 1–3): Elongate oval, sides subparallel, pygidium exposed beyond apices of elytra, apex of elytra broadly rounded. Head (Figs 16–18): Disc of frons and clypeus in lateral view nearly flat, clypeus with margins and apex reflexed. Frons and clypeus variably sculptured, punctate and/or rugose. Frontoclypeal suture weakly indicated, incomplete at middle. Eye canthus weakly cariniform. Interocular width 2.9–4.8 transverse eye diameters. Clypeal apex rounded or quadrate, with or without basolateral constriction, lacking bead; frontal view flat, length (at middle) about 1/3 length of frons, disc variably punctate and setose. Mandible (Fig. 4) with 1 apical, acute, recurved tooth; scissorial region with 1 poorly developed tooth; molar region narrow. Labrum projecting beyond clypeus, trapezoidal, apex emarginate or quadrate; apex moderately emarginate medially, surface moderately densely punctate, punctures moderate in size, some setose (setae moderately long and short, rufous). Maxilla (Fig. 5) lacking teeth; galea fused or not, with moderately dense, moderately long setae and with 1 to 2 long, bristle-like seta on disc; terminal segment of palpus with dorsal, longitudinal sulcus from based to sub-apex. Mentum (Figs 8–10) rectangular, trapezoidal, or pentagonal, inner apex produced anteriorly or not, with or without inner shelf. Antenna 9 or 10-segmented with 3-segmented club; club subequal in length to segments 1–7 combined or slightly longer than segments 1–7 combined. Pronotum: Widest at middle, apical angles obtuse, basal angles obtuse. Dorsal surface punctate, rugopunctate, or imbricate, with or without median line. Bead complete anteriorly, laterally, and basally. Margin sparsely setose (setae short, rufous). Scutellum: Parabolic, wider than long; base declivous at elytral base. Wing: Dense, thick setae present anterior to RA3+4 to apex; ScA with dense, thick setae near fold, lacking precostal pegs; AA1+2 shorter than AA3+4 (Fig. 7). Mesepimeron: Apex entirely hidden by base of elytra in dorsal and lateral views. Elytra: Variably sculptured with longitudinal, punctate striae; punctures variable. Sutural stria sulcate, impressed from base of scutellum to apex. Epipleuron from base to mid-metacoxa with shelf and associated setae; epipleuron from mid-metacoxa to apex beaded and with associated setae. Apex of elytra weakly rounded; sutural apex spiniform, rounded, or square. Elytral sutural length about 6.5 times length of scutellum. Propygidium: Hidden beneath elytra. Pygidium: Semitriangular, about twice as wide as long at middle; variably sculptured, punctate, shagreened, or weakly rugose. Margins beaded. Apex rounded. Apical bead with moderately long to long setae; setae tawny to rufous or testaceous. Venter: Prosternal keel triangular; apex projecting anteroventrally at about 35° with respect to ventral plane; apex produced to level of protrochanter, blunt; surface flat (lacking protuberance). Mesometasternal keel lacking. Sternites 1–4 subequal in length in male and female, sternites 5–6 about twice length of sternite 4. In lateral view, male sternites flat, female sternites weakly convex. Last sternite with apex quadrate in male, posteriorly rounded in female. Legs: Protibia (male and female) with width at base 1/3^rd^ to 1/4^th^ greater than width at apex (Figs 11–13), inner base with weak Protibial notch, with 3 external teeth in apical half (2 apical teeth removed from basal tooth external teeth or not); spur present, subapical. Modified foreclaw of male (Fig. 11) about 3 times width of unmodified claw, not toothed, inner apical tooth present at apex, small. Foreclaws of female simple, internal claw slightly wider than outer claw. Unguitractor plate laterally flattened, exposed beyond tarsomere 5; apex with 0–1 moderately long setae. Mesotibia with sides subparallel, apex weakly divergent; external edge with 2 carinae; inner apex with 2 spurs; apex with 10–20 spinulae. Meso- and metatarsomere 4 apicomedially with 4 medial spinulae (male) or 2 medial spinulae and 1 seta-like long spinule laterad of each medial spinula (female). Meso- and metatarsal claws of male and female simple, internal claw slightly wider than outer claw. Metarsomere 1 moderately divergent at middle and apex (male and female). Metatrochanter with apex weakly produced beyond posterior border of femur. Metacoxal corner (female) rounded or square. Metatibia (Figs 14–15) with sides subparallel, divergent towards apex; external edge with 1–2 carinae; inner apex with 2 spurs; inner apex with 25–40 short, stout spinulae. Spiculum gastrale: Y-shaped, lacking associated sclerites (Fig. 6). Parameres and phallobase: Plates fused dorsally/ventrally (not laterally). Dorsal plate symmetrical, apex rounded and with or without two apical, rounded teeth (Figs 19–21); not diagnostic for species. Ventral plate elongate (as long as dorsal plate or ½ length of dorsal plate), apex acute, quadrate, or rounded; diagnostic, species specific (Figs 19–21). Parameres slightly longer than phallobase. Female Genitalia: Gonocoxites subquadrate with sparse setae; not diagnostic for species.

##### Composition and distribution.

**Figure 22.**
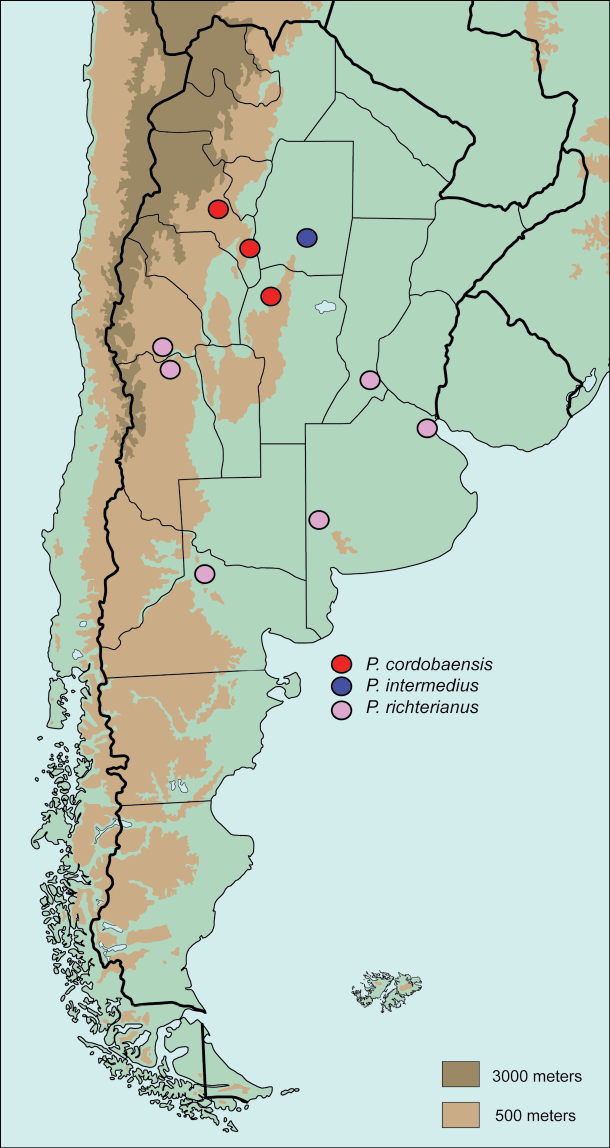
Distribution of *Pseudogeniates* species in Argentina

The genus *Pseudogeniates* is composed of three species that are distributed entirely in Argentina (Fig. 22). Species are associated with arid areas of the Pampa, Espinal, Chaco, and Monte eco-regions in Argentina.

##### Biology.

Very little is known of the biology of the species in the genus. Males and females are attracted to lights at night. Based on the extreme wear on the protibia of some specimens, individuals probably are associated with soil and use their front appendages for digging.

##### Etymology.

The name *Pseudogeniates* (“the false *Geniates*”) refers to the similarity in form to some species in the ruteline genus *Geniates*.

##### Key to species of *Pseudogeniates*

(Males: Protarsus with inner claw enlarged [Fig. 11]; abdomen ventrally flat or concave. Females: Protarsus with inner claw not enlarged, similar in size to outer claw [Figs 12–13]; abdomen ventrally swollen or weakly convex.)

**Table:** 

1	Mentum longer than wide, form subtrapezoidal or rectangular (Figs 9–10)	2
–	Mentum with length subequal to width, form pentagonal (Fig. 8)	*Pseudogeniates cordobaensis* Soula
2	Mentum with inner apex not projecting anteriorly (Fig. 9), with inner shelf; female metacoxal corner square; ventral plate of male parameres nearly as long as the dorsal plate (Fig. 20)	*Pseudogeniates intermedius* Ohaus
–	Mentum with inner apex projecting anteriorly (Fig. 10), without inner shelf; female metacoxal corner rounded; ventral plate of male parameres about half the length of the dorsal plate (Fig. 21)	*Pseudogeniates richterianus* Ohaus

#### Pseudogeniates cordobaensis

Soula, 2009

http://species-id.net/wiki/Pseudogeniates_cordobaensis

Figs 1 , 5, 6, 8 , 11, 14 , 16 , 19 , 22

Pseudogeniates cordobaensis Soula 2009: 122.

##### Material examined.

Holotype male, from Ohaus’s type series of *Pseudogeniates intermedius*, at ZMHB labeled: a) “Argentina S. d. Cordoba J. Hubrich S.” (typeset, white label), b) male symbol, c) “Pseudogeniates intermedius cotype Ohs.” (Ohaus’s handwritten, red label), d) “SYNTYPUS Pseudogeniates intermedius Ohaus, 1914 labelled by MNHUS 2007” (typeset, red label), f) “Paralectotype 2009 Pseudogeniates intermedius Oh. Soula det.” (typeset and handwritten, red label), g) “Holotype 2009 Pseudogeniates cordobaensis Soula Soula” (handwritten and typeset, red label). Soula (2009) based the description on one male specimen.

##### Description

(based on 10 males and 17 females). Length 13.8-17.0 mm. Widest width 6.8-8.0 mm. Color: Dorsum, venter, and appendages rufous-castaneous (Fig. 1). Head (Fig. 16): Frons densely punctate to confluently punctate (mid-disc), rugopunctate laterally and apically; punctures small and moderately large, mixed. Clypeus with dorsal surface densely punctate to confluently punctate (mid-disc), rugopunctate laterally and apically, punctures small and large (mixed), some setose; setae short to moderate, rufous, sparse; shape broadly semicircular, apex and sides broadly reflexed, with or without constriction basolaterally; disc (frontal view) densely rugopunctate, punctures small and large, mixed, some setose (setae short to moderately long, rufous, sparse). Labrum setose; setae moderately long and short, rufous. Maxilla (Fig. 5) with galea distinct, with 1 elongate, bristle-like, medial seta. Mentum (Fig. 8) nearly as long as wide or slightly longer than wide, form pentagonal, inner apex projects anteriorly, with inner shelf. Antenna 10-segmented with 3 segmented club; club slightly longer than stem. Pronotum: Medial line obsolete. Surface (disc and mediolaterally) moderately densely imbricate with sparse, short, testaceous setae. Margin with short, rufous setae. Elytra: Sutural stria impressed from base of scutellum to apex. Elytral apex weakly spiniform or quadrate. Discal striae weakly impressed, punctate; 5 on disc mesad of humerus, 5 laterad of humerus; discal stria 1 (adjacent to sutural stria) extending from base to apical umbone; striae 2-3 and 4-5 paired, extending from near base to apical umbone, stria 5 faintly impressed and incomplete; humeral striae 1-4 faintly impressed, incomplete, indicated by shallow punctures, stria 5 moderately impressed, extending from base of metacoxa to subapex; small to moderate in size, some longitudinal (Fig. 5). Intervals with moderately dense, random punctures, punctures minute to moderate in size; interval 1 broader than others. Pygidium: Disc and apex sparsely punctate; base and sides moderately densely imbricate, nearly obsolete; punctures minute to small. Legs: Protarsomere 5 of male subequal to tarsomeres 2-4. Modified proclaw of male subequal in length to tarsomeres 2-4; internoapical tooth present. Protibia (Fig. 11) (male and female) with teeth evenly separated; basal 1/3 weakly dilated (male) or moderately dilated (female). Mesotibia (male) with sides subparallel, weakly divergent towards apex (male) or weakly divergent (female); apex oblique with 10-11 moderately long spinulae; external edge with weak carina in basal 1/3, 1 carina in apical 1/3. Metatrochanter (male) weakly produced beyond posterior border of femur. Metacoxal corner square (female). Metacoxa at middle weakly produced posteriorly. Metatibia of male (Fig. 14) with sides subparallel, weakly divergent towards apex; external edge with 1 carina in basal 1/3 (faint), 1 carina in apical 1/3 (faint); apex without corbel, with 2 inner spurs (spurs equal in width in male, weakly curved at apex), inner apex with 28 short, stout spinulae. Metatibia of female divergent at apical 1/5; external edge with 1 carina in basal 1/3, 1 carina in apical 1/3; apex without corbel, with 2 inner spurs, inner apex with 28 short, stout spinulae. Parameres: Ventral plate nearly as long as dorsal plate with sides converging to a quadrate apex (Fig. 19).

##### Diagnosis.

*Pseudogeniates cordobaensis* is separated from other species in the genus by the pentagonal form of the mentum (width subequal to length) that has the inner apex projecting anteriorly and has an inner shelf (Fig. 8). In comparison, the form of mentum is longer than wide in *Pseudogeniates intermedius* and *Pseudogeniates richterianus* (Figs 9-10). In *Pseudogeniates intermedius*, the inner apex of the mentum does not project anteriorly, but does possess an inner shelf (Fig. 9); in *Pseudogeniates richterianus*, the inner apex projects anteriorly, but does not possess an inner shelf (Fig. 10). The ventral plate of the male parameres in *Pseudogeniates cordob-aensis* is nearly as long as dorsal plate with sides converging to a quadrate apex (Fig. 19). The ventral plate of *Pseudogeniates richterianus* is about half the length of the dorsal plate (Fig. 21), whereas in *Pseudogeniates intermedius* the ventral plate is nearly as long as the dorsal plate, but the sides converge with a weak constriction preapically and a rounded apex (Fig. 20).

##### Distribution

**(Fig. 22).** *Pseudogeniates cordobaensis* is distributed in the Monte de Sierras y Bolsones in the Monte eco-region in Argentina. The distribution of this species coincides with the Montane Forest region (Navarro et al. 2009) and the Yungas Forest area of endemism in Argentina (Szumik et al. 2012).

##### Locality data.

27 specimens from IAZA, WSUC, and ZMHB. **ARGENTINA** (27): CATAMARCA (26): Salar de Pipanaco, Pío Brizuela (37 km S Andalgalá, 27°49'34"S, 66°14'47"W, 751 m), Quirós (2 km S on RN 157, 28°48'43.3"S, 65°06'22.6"W), CÓRDOBA (1): Huerta Grande (Sierra de Córdoba).

##### Temporal data.

December (9).

##### Remarks.

The holotype specimen for this species was part of the type series for *Pseudogeniates intermedius*, a series that included three specimens from Santiago del Estero and one specimen (=*Pseudogeniates cordobaensis*) from Huerta Grande in the Sierra de Cordóba, Córdoba Province (Ohaus 1914).

##### Natural history.

This species is attracted to black lights. It was recorded from an elevation of 751 m.

#### Pseudogeniates intermedius

Ohaus, 1914

http://species-id.net/wiki/Pseudogeniates_intermedius

Figs 2 , 9 , 17 , 20

Pseudogeniates intermedius Ohaus 1914: 303.

##### Material examined.

Type material (3 males, 1 female) at ZMHB. Lectotype male labeled: a) “ARGENTINA Santiago d E. Wagner” (typeset, white label), b) male genitalia card-mounted, c) mouthparts card-mounted, d) “Typus!” (typeset, red label), e) “Pseudogeniates intermedius Ohs.” (Ohaus’ handwritten, red label), f) “SYNTYPUS Pseudogeniates intermedius Ohaus, 1914 labelled by MNHUS 2007” (typeset, red label), g) “Lectotype 2009 Pseudogeniates intermedius Oh. Soula” (typeset and handwritten, red label). Paralectotype female labeled: a) “ARGENTINA Santiago del Estero” (typeset, white label), b) female symbol, c) mouthparts card-mounted, d) “Pseudogeniates intermedius cotype Ohs.” (Ohaus’ handwritten, red label), e) “SYNTYPUS Pseudogeniates intermedius Ohaus, 1914 labelled by MNHUS 2007” (typeset, red label), f) “Paralectotype 2009 Pseudogeniates intermedius Oh. Soula det.” (typeset and handwritten, red label), g) “Alloréférent Pseudogeniates intermedius M. SOULA det 19” (handwritten and typeset, white label with scribed red boarder), h) “Paralectotype 2009 Pseudogeniates intermedius Oh. Soula det.” (typeset and handwritten, red label). A second male paralectotype labeled: a) “MUSEUM PARIS PROV. DE SANTIAGO del Estero Barrancas Banados de Rio Dulce, 80 kil. O. d’Icano E.R. WAGNER 1909”, b) “Pseudogeniates intermedius cotype Ohs.” (Ohaus’ handwritten, red label), c) “SYNTYPUS Pseudogeniates intermedius Ohaus, 1914 labelled by MNHUS 2007” (typeset, red label), d) “Paralectotype 2009 Pseudogeniates intermedius Oh. Soula det.” (typeset and handwritten, red label). An additional paralectotype from Córdoba is the holotype specimen for *Pseudogeniates cordobaensis* Soula (see treatment for *Pseudogeniates cordobaensis*). Ohaus (1914) stated that he described the species based on four specimens: two males and one female from Santiago del Estero (Wagner collection) and one male from Sierra de Córdoba.

##### Description

(based on 2 males and 1 female). Length 14.0–15.7 mm. Widest width 7.5–9.0 mm. Color: Dorsum, venter, and appendages rufotestaceous to castaneous (Fig. 2). Head (Fig. 17): Frons densely, confluently punctate or rugopunctate; punctures small and moderately large, mixed. Clypeus with dorsal surface densely punctate, confluently punctate or rugopunctate, punctures small and large, mixed; clypeal shape broadly semicircular, apex and sides broadly reflexed, with or without constriction basolaterally; disc (frontal view) with surface densely rugopunctate, punctures small and large, mixed, some setose (setae short to moderately long, rufous, sparse). Labrum with setae moderately long and short, rufous. Maxilla with galea fused, with 1 or 2 elongate bristle-like, medial, setae. Mentum (Fig. 9) longer than wide, form subtrapezoidal (base broader than apex), inner apex not projecting anteriorly, with inner shelf. Antenna 10-segmented with 3 segmented club; club subequal to stem. Pronotum: Medial line obsolete (male) or weakly indicated (female). Surface (disc) moderately densely imbricate with sparse, short, testaceous setae; surface (mediolaterally) moderately densely rugopunctate, punctures minute and small. Margin with setae short, testaceous. Elytra: Elytral apex rounded or quadrate (not spiniform). Discal striae punctate; 5 on disc mesad of humerus, 5 laterad of humerus; discal stria 1 (adjacent to sutural stria) extending from base to apical umbone; striae 2-3 and 4-5 paired, extending from near base to apical umbone; humeral stria 1 incomplete (extending from mid-elytron to near apical umbone), stria 2 extending from humerus to near apical umbone, striae 3-4 extending from base of metacoxa to near apical umbone (indicated by punctures, not sulcate), stria 5 poorly indicated, extending from base of metacoxa to subapex (indicated by punctures near base, sulcate near apex); punctures small, some longitudinal. Intervals with moderately dense, random punctures, some transversely wrinkled (on disc), punctures small; interval 1 broader than others. Pygidium: Disc and apex moderately densely punctate, some transverse; base and sides closely, weakly rugulose; punctures minute to small. Legs: Protarsomere 5 of male subequal to tarsomeres 2-4. Modified foreclaw of male subequal in length to tarsomeres 2-4; internoapical tooth present. Protibia (male and female) with external teeth evenly separated; basal 1/3 weakly dilated (male) or moderately dilated (female) (e.g., Figs 11-13). Mesotibia (male) with sides subparallel, weakly divergent towards apex; apex oblique with 8-11 moderately long spinulae; external edge with weak carina in basal 1/3, 1 carina in apical 1/3. Metatrochanter (male and female) weakly produced beyond posterior border of femur. Metacoxal corner square (female). Metacoxa at middle weakly produced posteriorly. Metatibia (male) with sides subparallel, apex weakly divergent; external edge with 1 carina in basal 1/3, 1 carina in apical 1/3; apex without corbel, with 2 inner spurs (spurs equal in width in male, with a weak curve at apex), inner apex with 21-25 short, stout spinulae. Metatibia (female) greatly divergent at apical 1/5; external edge with 1 carina in basal 1/3, 1 carina in apical 1/3; apex without corbel, with 2 inner spurs, inner apex with 28 short, stout spinulae. Parameres: Ventral plate nearly as long as dorsal plate but the sides converge with a weak constriction preapically and a rounded apex (Fig. 20).

##### Diagnosis.

*Pseudogeniates intermedius* is known from only three specimens. It is separated from other species in the genus by the form of the mentum (Fig. 9) and the form of the male parameres (Fig. 20). It is distinguished from *Pseudogeniates cordobaensis* by the form of the mentum (longer than wide and subtrapezoidal in *Pseudogeniates intermedius* [Fig. 9]; length subequal to width and pentagonal in *Pseudogeniates cordobaensis* [Fig. 8]) and apex of the ventral plate of the male parameres (with a weak constriction preapically and a rounded apex in *Pseudogeniates intermedius* [Fig. 20]; lacking preapical constriction and with quadrate apex in *Pseudogeniates cordobaensis* [Fig. 19]). It is separated from *Pseudogeniates richterianus* by the apex of the mentum (with an inner shelf in *Pseudogeniates intermedius*; lacking inner shelf in *Pseudogeniates richterianus* [Fig. 9 versus Fig. 10]) and length of the ventral plate of the male parameres (nearly as long as dorsal plate in *Pseudogeniates intermedius* [Fig. 20]; half length of dorsal plate in *Pseudogeniates richterianus* [Fig. 21]).

##### Distribution

(Fig. 22). *Pseudogeniates intermedius* is distributed in the Chaco-seco eco-region in Argentina.

##### Locality data.

3 specimens from ZMHB. **ARGENTINA** (3): SANTIAGO DEL ESTERO (3): Barrancas (Bañados de Río Dulce, 80 km W. d’Icaño), No data.

##### Temporal data.

December (1).

##### Natural history.

This species is known from two male specimens and one female specimen, and the natural history is not known.

##### Remarks.

Ohaus (1914) distinguished *Pseudogeniates intermedius* from *Pseudogeniates richterianus* based on the 10-segmented antenna (versus 9-segmented in *Pseudogeniates richterianus*), the “front corners” of the clypeus (rounded in *Pseudogeniates richterianus* versus absent in *Pseudogeniates intermedius*), and elytral coloration (brownish-red color in *Pseudogeniates intermedius* versus reddish-yellow in *Pseudogeniates richterianus*). Based on our examination of specimens, these characters are highly variable and are not reliable for identification. Coloration varies within species (from testaceous to castaneous), form of the clypeus varies, and antennae vary from 9 to 10 segments. However, characters provided in our “Diagnosis” are sufficient to separate *Pseudogeniates intermedius* and *Pseudogeniates richterianus*.

#### Pseudogeniates richterianus

Ohaus, 1910

http://species-id.net/wiki/Pseudogeniates_richterianus

Figs 3 –4, 7, 10 , 12–13, 15 , 18 , 21 –22

Pseudogeniates richterianus Ohaus 1910: 180.
Pseudogeniates richteri Ohaus (*lapsus* in Ohaus 1934, table 2, figure 6).

##### Material examined.

Two female co-types examined at ZMHB. Female lectotype labeled: a) “ARGENTINA Buenos Aires XII.08 H.R.” (typeset and handwritten on upperside and underside of white label), b) “Pseudogeniates Richteri cotype Ohs.” (Ohaus’ handwritten, red label), c) “SYNTYPUS Pseudogeniates richterianus Ohaus, 1910 labelled by MNHUB 2007” (typeset, red label), d) “Lectotype 2009 Pseudogeniates Richterianus Oh. Soula” (typeset and handwritten, red label). Paralectotype female labeled: a) “Rep. Argentina Prov. Santa Fe, R. Richter”, b) female symbol, c) mouthparts card-mounted, d) “Typus!” (typeset, red label), e) “Pseudogeniates Richterianus Oh.” (Ohaus’ handwritten, red label), f) “SYNTYPUS Pseudogeniates richterianus Ohaus, 1910 labelled by MNHUB 2007” (typeset, red label), d) “Lectotype 2009 Pseudogeniates Richterianus Oh. Soula det.” (typeset and handwritten, red label). The paralectotype has the head, thorax, and abdomen glued together. It is, in fact, a chimera with the head of *Parhomonyx fuscoaneus* and body of *Pseudogeniates richterianus*. Ohaus (1910) stated that he described the species based on three female specimens from Santa Fé, Argentina (Hans Richter collection) and Buenos Aires. The location of one specimen is not known.

##### Description

(based on 4 males and 22 females). Length 12.0–18.4 mm. Widest width 7.8–10.0 mm. Color: Dorsum, venter, and appendages rufotestaceous to castaneous (Fig. 3). Head (Fig. 18): Frons moderately densely punctate or rugopunctate; punctures small and moderately large, mixed. Clypeus with dorsal surface weakly rugopunctate, punctures small and large, mixed; shape broadly rounded apically and laterally or subquadrate, with or without constriction basolaterally, apex and sides broadly reflexed; disc (frontal view) moderately densely punctate or rugopunctate, punctures small and large, mixed, some setose (setae short, testaceous, sparse). Labrum with setae moderately long and short, testaceous. Maxilla with galea not fused, with 1-2 moderately long, bristle-like medial setae. Mentum (Fig. 10) longer than wide, rectangular, inner apex projecting anteriorly, without inner shelf. Antenna 9 or 10-segmented with 3-segmented club; club subequal to or slightly longer than stem. Pronotum: Medial line weakly impressed or obsolete. Surface (disc) moderately densely punctate (mid-disc), rugopunctate, or imbricate; punctures small to moderate. Margin with setae moderately long, testaceous. Elytra: Elytral apex rounded, quadrate, or with obtuse angle. Discal stiae, sulcate-punctate; 5 on disc mesad of humerus, 5 laterad of humerus; discal stria 1 (adjacent to sutural stria) extending from base to apex, stiae 2-6 extending from near base to apical umbone; humeral stria 1 incomplete (extending from mid-elytron to near apical umbone), stria 2 extending from humerus to near apical umbone, striae 3-4 extending from base of metacoxa to near apical umbone (indicated by punctures, not sulcate), stria 5 extending from base of metacoxa to subapex (indicated by punctures near base, sulcate near apex); punctures small to moderate in size, some longitudinal. Intervals with moderately dense, random punctures, some transversely wrinkled (on disc), punctures small to moderate in size; interval 1 subequal in width to other intervals. Pygidium: Disc and apex moderately densely punctate or transversely rugopunctate; base and sides closely, rugopunctate or weakly rugose; punctures small. Legs: Protarsomere 5 of male slightly longer than tarsomeres 2-4. Modified foreclaw of male slightly longer than tarsomeres 2-4; internoapical tooth present. Protibia with external teeth often worn; 2 apical teeth removed from basal tooth; basal 1/3 weakly dilated (male; Fig. 12) or moderately dilated (female; Fig. 13). Mesotibia with sides subparallel, apex weakly divergent and weakly oblique; external edge with weak carina in basal 1/3, 1 carina in apical 1/3; apex with 8-14 short spinulae. Metatrochanter weakly produced beyond posterior border of femur. Metacoxal corner (female) rounded, with or without inner tooth. Metacoxa at middle not produced posteriorly. Metatibia (male) with sides subparallel, apex moderately divergent in apical 1/3; external edge with 1 carina in basal 1/4, 1 carina in apical 1/4; apex without corbel, with 2 inner spurs (spurs equal in width in male), inner apex with 28-35 short, stout spinulae. Metatibia (female; Fig. 15) greatly divergent at apical 1/4; external edge with 1 carina in basal 1/3, 1 carina in apical 1/3; apex without corbel, with 2 inner spurs, inner apex with 28-35 short, stout spinulae. Parameres: Ventral plate half length of dorsal plate (Fig. 21).

##### Diagnosis.

*Pseudogeniates richterianus* is a highly variable species. Variation is observed in the antenna (9- or 10-segmented), labrum (weakly or moderately emarginated), length of antennal club (subequal to slightly longer then the stem), elytral apex (spiniform, quadrate, rounded, or obtusely angled), pronotal medial line (weak or obsolete), and form of the clypeus. However, several characters reliably separate this species from others in the genus. *Pseudogeniates richterianus* is separated from *Pseudogeniates intermedius* and *Pseudogeniates cordobaensis* by the form of the mentum (rectangular and without inner shelf in *Pseudogeniates richterianus* [Fig. 10]; subtrapezoidal and with inner shelf in *Pseudogeniates intermedius* [Fig. 9]; pentagonal and nearly as wide as long in *Pseudogeniates cordobaensis* [Fig. 8]), ventral plate of the parameres (half length of the dorsal plate in *Pseudogeniates richterianus* [Fig. 21]; nearly as long as dorsal plate in *Pseudogeniates intermedius* and *Pseudogeniates cordobaensis* [Figs 20 and 19, respectively]), metacoxal corner in female (rounded in *Pseudogeniates richterianus*; square in *Pseudogeniates intermedius* and in *Pseudogeniates cordobaensis*), and posterior margin of the metacoxa (not produced posteriorly in *Pseudogeniates richterianus*; produced posteriorly in *Pseudogeniates intermedius* and *Pseudogeniates cordobaensis*).

##### Distribution

(Fig. 22). *Pseudogeniates richterianus* is the most wide-spread species in the genus. It is distributed in the Pampa, Espinal, and Monte de Llanuras y Mesetas (Monte) eco-regions in Argentina.

##### Locality data.

26 specimens deposited in CMNC, FMNH, IAZA, MACN, MLJC, UCCC, and ZMHB. **ARGENTINA** (26): BUENOS AIRES (16): Caballito, Dept. Puán, No data. MENDOZA (7): Lavalle (Brazo S. Río San Juan Area San Miguel 32°20'7"S, 68°26'57.9"W, 500 m), San Rafael, No data. NEUQUEN (1): No data. RIO NEGRO (1): Villa Regina. SAN JUAN (1): Los Berros (Dept. Sarmiento). SANTA FE (1): Rosario.

##### Temporal data. 

November (5), January (3), February (1).

##### Natural history.

Based on specimens in collections, there is a female sex bias in this species (24 females: 4 males). Many specimens (male and female) have worn protibiae (Fig. 13), indicating that adults dig in abrasive soil. Label data indicate that this species was collected at mercury vapor light and at 500 m elevation.

##### Remarks.

Ohaus (1914) distinguished *Pseudogeniates richterianus* from *Pseudogeniates intermedius* based characters that vary within the species (see “Remarks” for P.* intermedius*), including number of antennal segments, form of the clypeus, and coloration. Although these characters are unreliable for diagnosis of the species, we provide characters that are useful (see “Diagnosis”). Ohaus (1910) named the species in honor of Herr Hans Richter from Buenos Aires.

## Discussion

Species in the genus *Pseudogeniates* exhibit a great deal of character variation, thus causing historical difficulty with circumscription of the species. Variation in the number of antennomeres, length of the antennal club, and form of the clypeal apex, labrum, elytral apex exhibit intraspecific variability. Variation of this degree is not unprecedented within the Scarabaeoidea. In particular, species associated with high elevations (e.g., *Parabyrsopolis* Ohaus) and species associated with arid habitats (e.g., *Anomiopsoides heteroclyta* (Blanchard), *Eucranium arachnoides* Brullé [both Scarabaeinae], and *Allidiostoma hirtum* Ohaus [Allidiostomatinae]) are known to possess broad intraspecific variation (Jameson 1990, Ocampo 2007). In some populations, individuals of *Parabyrsopolis chihuahuae* (Bates) exhibit a wide range in clypeal shapes (quadrate or parabolic, reflexed or not) (Jameson 1990). Individuals of *Allidiostoma heteroclyta* exhibit high variability in clypeal shapes and clypeal processes, as well as variation in pronotal sculpture (puncture shape and density) (Ocampo 2007). Species in the Mexican genus *Parachrysina* Bates are unusual in that some species have 8-segmented antennae and others have 9-segmented antennae (Jameson 1991). Molecular analysis of species of *Pseudogeniates*, as well as other highly variable species, may reveal underlying mechanisms for high intraspecific variation.

High intraspecific variation may have been the product of historical climatic and concomitant habitat fluctuations. During the Pleistocene, climatic fluctuations in northern Argentina may have resulted in broad regions being inhabited by Yungas forests (reaching to Córdoba province in the south) (Navarro et al. 2009). Subsequently, these forests have been replaced with remnant patches of Yungas, Chaco, and Espinal forests (Navarro et al. 2009). Climatic fluctuations and changing habitats, in combination with the latitudinal and altitudinal gradient of the montane region (Barquez and Díaz 2001), may have assisted in isolating populations (such as ancestral populations of *Pseudogeniates*), influencing species diversifications, and leading to high levels of endemism (Navarro et al. 2009, Szumik et al. 2012).

## Supplementary Material

XML Treatment for Pseudogeniates

XML Treatment for Pseudogeniates cordobaensis

XML Treatment for Pseudogeniates intermedius

XML Treatment for Pseudogeniates richterianus

## Appendix

*Pseudogeniates* locality table. (doi: 10.3897/zookeys.241.3802.app) File format: Microsoft Excel document (xls). **Explanation note:** Distribution maps were generated by entering latitude and longitude data into Microsoft Excel 2008 and uploaded to EarthPoint (http://www.earthpoint.us/ExcelToKml.aspx) and GoogleEarth (http://www.google.com/earth/index.html). This supplementary file allows addition of data and interactive mapping or niche modeling.
